# Association between genetically predicted leukocyte telomere length and non-scarring alopecia: A two-sample Mendelian randomization study

**DOI:** 10.3389/fimmu.2022.1072573

**Published:** 2023-01-30

**Authors:** Yicheng Li, Shuting Yang, Minjun Liao, Zijun Zheng, Mengyao Li, Xuerong Wei, Mengqian Liu, Lei Yang

**Affiliations:** ^1^ Department of Burns, Nanfang Hospital, Southern Medical University, Guangzhou, China; ^2^ National Clinical Research Center for Metabolic Diseases, Key Laboratory of Diabetes Immunology (Central South University), Ministry of Education, and Department of Metabolism and Endocrinology, The Second Xiangya Hospital of Central South University, Changsha, Hunan, China; ^3^ Guangdong Provincial Key Laboratory of Gastroenterology, Department of Gastroenterology and Hepatology Unit, Nanfang Hospital, Southern Medical University, Guangzhou, China

**Keywords:** androgenetic alopecia, alopecia areata, non-scarring alopecia, Mendelian randomization, leukocyte telomere length

## Abstract

**Background:**

The most commonly acknowledged non-scarring alopecia are androgenetic alopecia (AGA) and alopecia areata (AA). Previous studies have revealed various risk factors associated with alopecia. However, the relationship between leukocyte telomere length (LTL) and non-scarring alopecia remains unclear.

**Methods:**

A two-sample Mendelian randomization (MR) analysis was performed to evaluate the causality between genetically predicted LTL and the risk of non-scarring alopecia. MR analyses were performed using the inverse variance-weighted (IVW) method and complemented with other MR methods.

**Results:**

The summary statistics of the genome-wide association studies (GWAS) for AGA and AA were obtained from the FinnGen biobank, which included 119,185 and 211,428 individuals, respectively. A total of 126 single nucleotide polymorphisms (SNPs) with genome-wide significance were selected as the instrumental variables for LTL. The MR analyses suggested a causal relationship between LTL and AGA, and the risk of AGA increased by 3.19 times as the genetically predicted LTL was shortened by one standard deviation in log transformed form under the IVW method (OR = 4.19, 95% CI = 1.20–14.61, *p* = 0.024). The other MR methods also demonstrated a similar trend of the effect of LTL on AGA. There was no causal relationship between LTL and AA (*p* > 0.05). Sensitivity analyses further demonstrated that the current results were less likely to be affected by confounders and bias.

**Conclusion:**

Our results suggested a potential causal relationship between LTL and AGA, and shortened LTL was associated with an increased risk of AGA.

## Introduction

The two most commonly known non-scarring alopecia are androgenetic alopecia (AGA) and alopecia areata (AA). Although known as male pattern baldness (MPB), AGA is one of the most common types of chronic hair loss for both sexes, which affects at least 80% of men and half of women at the age of 70 years, with the incidence increasing with age (1, 2). AGA is characterized by an alteration of the hair growth cycle, which includes a reduced anagen phase duration and an increased telogen phase duration, resulting in gradual transformation of terminal hair into intermediate or vellus hair and, finally, balding. The most common forms of AGA consist of the representative receding of the frontal hairline for men together with the thinning of the hair between the frontal and the vertex scalp, but a maintained frontal hairline for women (2–4).

AA is a chronic hair follicle-specific autoimmune disorder characterized by patches of non-scarring alopecia, which influences approximately 2% of the population (5, 6). Nevertheless, the exact mechanisms remain under investigation, but a common feature is the collapse of the immune privilege of the hair follicles induced by immunological mechanisms (7). Telogen effluvium (TE) is also one of the important subtypes of non-scarring alopecia (8). However, due to the lack of existing summary data from the results of genome-wide association studies (GWAS), we excluded TE from this study.

Complicated genetic susceptibility, oxidative stress injury, environmental factors, lifestyle, and advanced age cooperatively contribute to the occurrence and severity of hair loss, among which, aging may play an important role in its pathological changes. Telomeres are the special nucleoprotein structures located at the ends of linear chromosomes that could protect their integrity (9, 10). The leukocyte telomere length (LTL) is shortened gradually with cellular divisions, and gene mutations or DNA damage sometimes occurs during this process, resulting in alterations in the cellular life span and senescence (11). Therefore, LTL has become widely recognized as a measure of an individual’s biological age and as a biomarker for certain age-related diseases (12). Previous observational studies have revealed some risk factors for alopecia; however, the relationship between aging and non-scarring alopecia has not been well studied. Therefore, we applied the Mendelian randomization (MR) method to determine whether there is a causal relationship between LTL and the two main subtypes of non-scarring alopecia.

MR is a method of instrumental variable (IV) analysis that uses single nucleotide polymorphisms (SNPs) from GWAS as instruments, which are representative of the exposure characteristics, to reveal causal relationships between complex characteristics, provided that the necessary assumptions have been satisfied (13). IVs are not correlated with random error (existence of deviation, such as age, sex, and weight, which need to be matched in observational studies) because SNP allocation follows the Mendelian inheritance rule, suggesting random allocation during the formation of a fertilized ovum. Therefore, based on SNP acting as the IV, the basic principle of MR research is comparable to that of randomized controlled trials (RCTs). This strategy is relatively convenient, cost-effective, and less likely to be confounded by covariables (14–17). Therefore, in this study, we used available summary statistics from open-access GWAS databases to perform a two-sample MR analysis and evaluated the causality between LTL and non-scarring alopecia. The results of the current study may partly explain the underlying mechanism of non-scarring alopecia and may offer support in the future development of prevention and intervention strategies.

## Materials and methods

### Mendelian randomization study design

**Figure 1:**
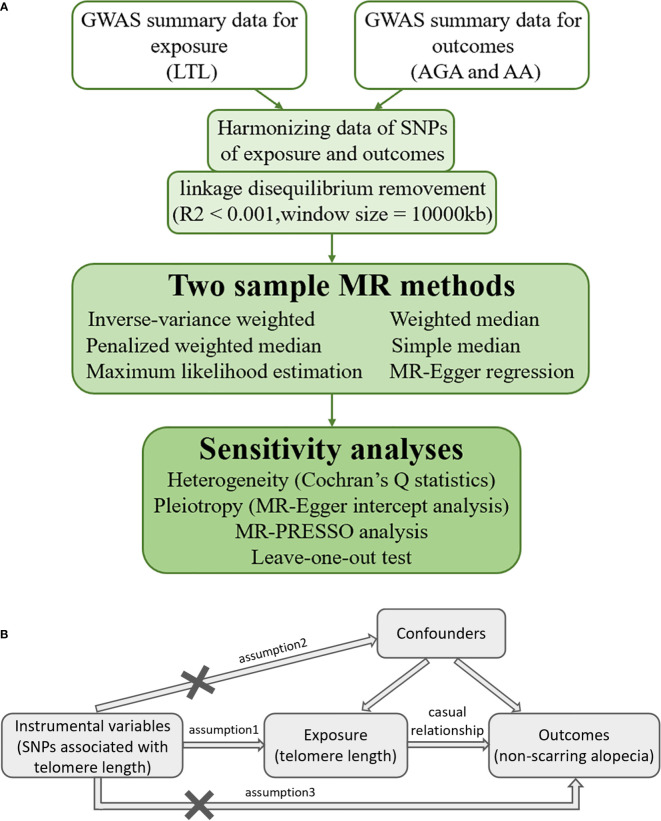
**(A)** Detailed flowchart of the current two-sample Mendelian randomization study. **(B)** Mendelian randomization assumptions. *GWAS*, genome-wide summary association study; *LTL*, leukocyte telomere length; *AGA*, androgenetic alopecia; *AA*, alopecia areata; *SNP*, single nucleotide polymorphism; *MR*, Mendelian randomization; *MR-Egger*, Mendelian randomization-Egger; *MR-PRESSO*, Mendelian randomization pleiotropy residual sum and outlier.

MR is a statistical method used to evaluate causality without potential bias caused by confounders (18). Genetic variant is the most important and effective IV in an MR study. Eligible IVs should satisfy three important assumptions according to the MR theory: 1) the IVs are strongly correlated with the interested exposure factor; 2) the IVs follow the rule of random assignment and do not affect the outcome through confounders; and 3) the IVs do not exert influence on the outcome directly, but indirectly through the hypothesized causal pathway of exposure under investigation (19). The detailed flowchart of this MR study is shown in **Figure 1A**, while the MR assumptions are depicted in **Figure 1B**.

In this study, we selected SNPs closely associated with LTL as the IVs using summary data from open-access GWAS databases to evaluate the potential causal effect of the exposure (i.e., LTL) on the outcomes (i.e., AGA and AA). Moreover, the data for exposure and outcomes were obtained from different independent samples. Ethical approval was provided in the preliminary studies and was no longer required for this study.

### Instrument variable selection

The summary statistics were obtained from the European Network for Genetic and Genomic Epidemiology (ENGAGE), which involved 472,174 European participants in the GWAS project for LTL. Quantitative polymerase chain reaction (PCR) analyses were performed to obtain LTL measurements for these participants from the UK Biobank (UKB) (9). The UKB recruited a prospective cohort of more than 500,000 volunteers aged 40–69 years (20). A total of 197 SNPs showed independent associations with LTL, as identified by the above-mentioned GWAS meta-analysis.

SNPs with genome-wide significance were selected as the IVs of telomere length for further study (*p* < 5 × 10^8^). Linkage disequilibrium (LD) was used to determine whether the SNPs were genetically linked or not, and the threshold was set as *r* ^2^ < 0.001, with a window size of 10,000 kb. SNPs associated with potential confounding factors of AGA and AA were removed from the final MR analyses according to the PhenoScanner V2 database (http://www.phenoscanner.medschl.cam.ac.uk/), an open-access database for genotype–phenotype associations. The palindromic variants were also excluded from this study, and the directions of the effects of SNPs on LTL and AGA, as well as AA, were harmonized. Eventually, a total of 126 eligible SNPs were included for the final MR analyses. Information on these SNPs is detailed in **Supplementary Table S1**.

### Summary data sources for the subtypes of non-scarring alopecia

The aggregated data of the GWAS for AGA and AA were obtained from the open-access GWAS website (https://gwas.mrcieu.ac.uk/), with 119,185 and 211,428, respectively, European participants from the FinnGen biobank. The mean age at the first event was 47.7 years for AGA and 41.9 years for AA. The data for AGA and AA were obtained from participants from another independent consortium different from those in the GWAS for LTL. However, these SNPs were all derived from GWAS on European ancestry to minimize potential bias caused by population heterogeneity.

### Statistical analyses of the MR study

For this study, the inverse variance-weighted (IVW), the Mendelian randomization-Egger (MR-Egger), the weighted median, the penalized weighted median, the simple median, and the maximum likelihood estimation methods were adopted to evaluate the risk association between shortened LTL and AGA, as well as AA (15, 21). Of the above methods, IVW is the most commonly used variant-specific causal estimations in a two-sample MR analysis, especially when all the enrolled IVs were of robust validity. Therefore, we adopted IVW as the primary and most efficient analysis method in this study (22). When heterogeneity was statistically significant in the research studies, a random effects IVW model was applied; otherwise, a fixed effects IVW model was adopted.

Other supplementary methods were also used for validation of the consistency and efficiency of the MR results. The MR-Egger regression is mainly used to evaluate horizontal pleiotropy and may provide causal estimation in the case of weaker IVs (23, 24). The penalized weighted median estimation is a new analytic method modified from the original weighted median method; thus, the IVs substantially contributing to the heterogeneity would be penalized, but the results would still be reliable provided that at least half of the IVs were valid (25). The maximum likelihood method was used to estimate the probability distribution by maximizing the likelihood function with low standard errors (26). Furthermore, the simple median method has also been applied in many other studies (21, 27). Moreover, scatter, forest, and funnel plots were drawn to visualize the results and the efficiency and stability of the MR study.

### Sensitivity analyses

Sensitivity analyses were performed, including heterogeneity, pleiotropy, leave-one-out tests, and the Mendelian randomization pleiotropy residual sum and outlier (MR-PRESSO). The heterogeneity of the IVs applied in the IVW method was assessed using Cochran’s *Q* test. A *p*-value smaller than 0.05 was considered to suggest statistically significant heterogeneity. And the MR result should be carefully interpreted under this circumstances. Pleiotropy refers to a single locus possibly affecting multiple phenotypes. The MR-Egger intercept analysis was applied to evaluate directional pleiotropy, and the pleiotropy effect could be ignored when *p* > 0.05 (24). In addition, the MR-PRESSO analysis was performed to assess the influence of outliers. The leave-one-out test was performed by removing the SNPs one by one. When there is no great change to the remaining results from such a removal process (the result lines remained on the same side of the zero point), the causal relationship is stable and reliable.

All statistical analyses were performed using the “Two-Sample MR” and “MRPRESSO” packages in R software (version 4.1.1).

## Results

### Genetically predicted shorter LTL is associated with increased risk of AGA instead of AA

**Figure 2:**
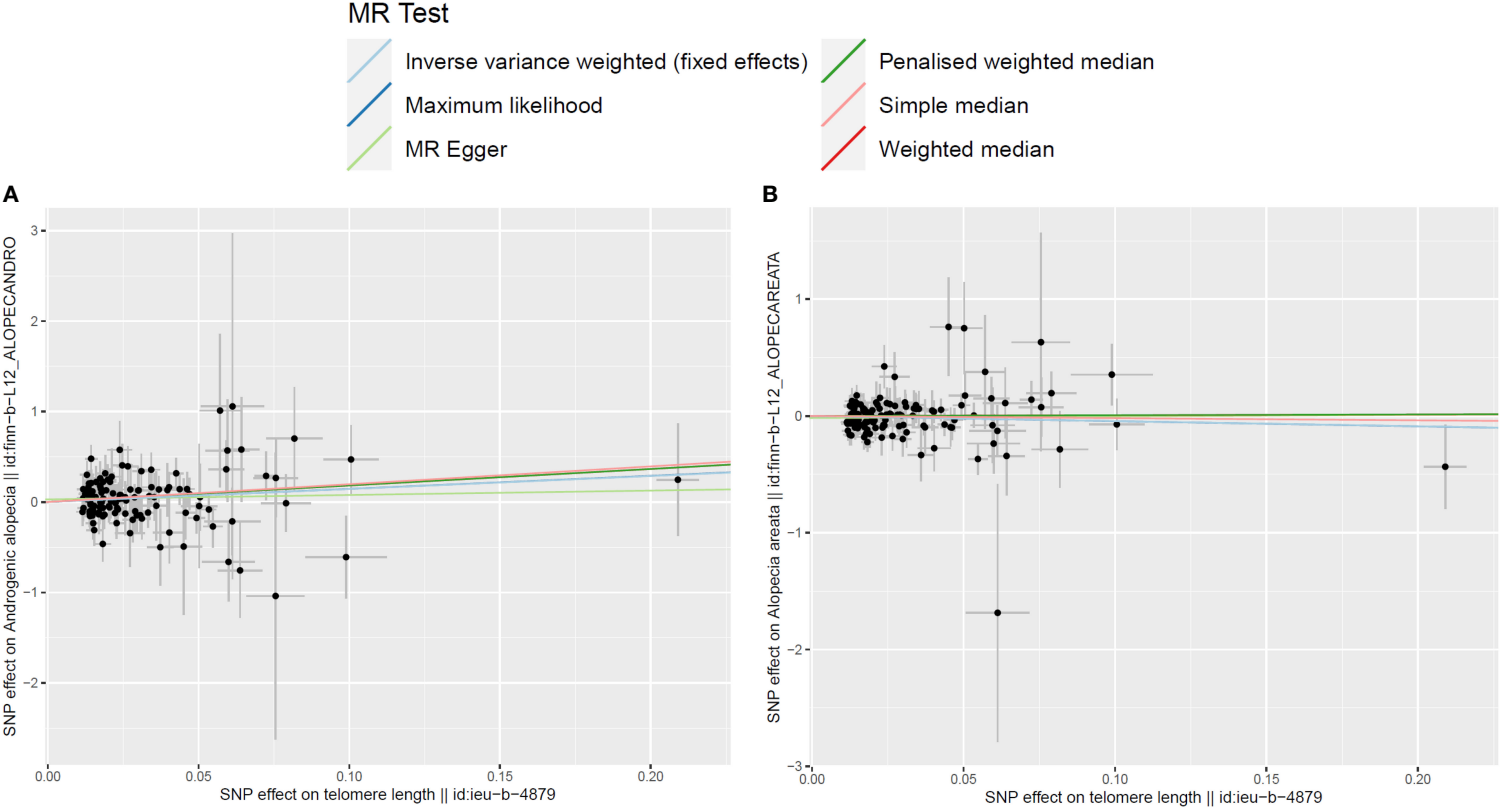
Scatter plots for the effects of single-nucleotide polymorphisms (SNPs) on telomere length and non-scarring alopecia. The x-axis represents the effects of each genetic variant on telomere length, and the y-axis represents the effects of each genetic variant on androgenetic alopecia **(A)** or alopecia areata **(B)**.

**Figure 3:**
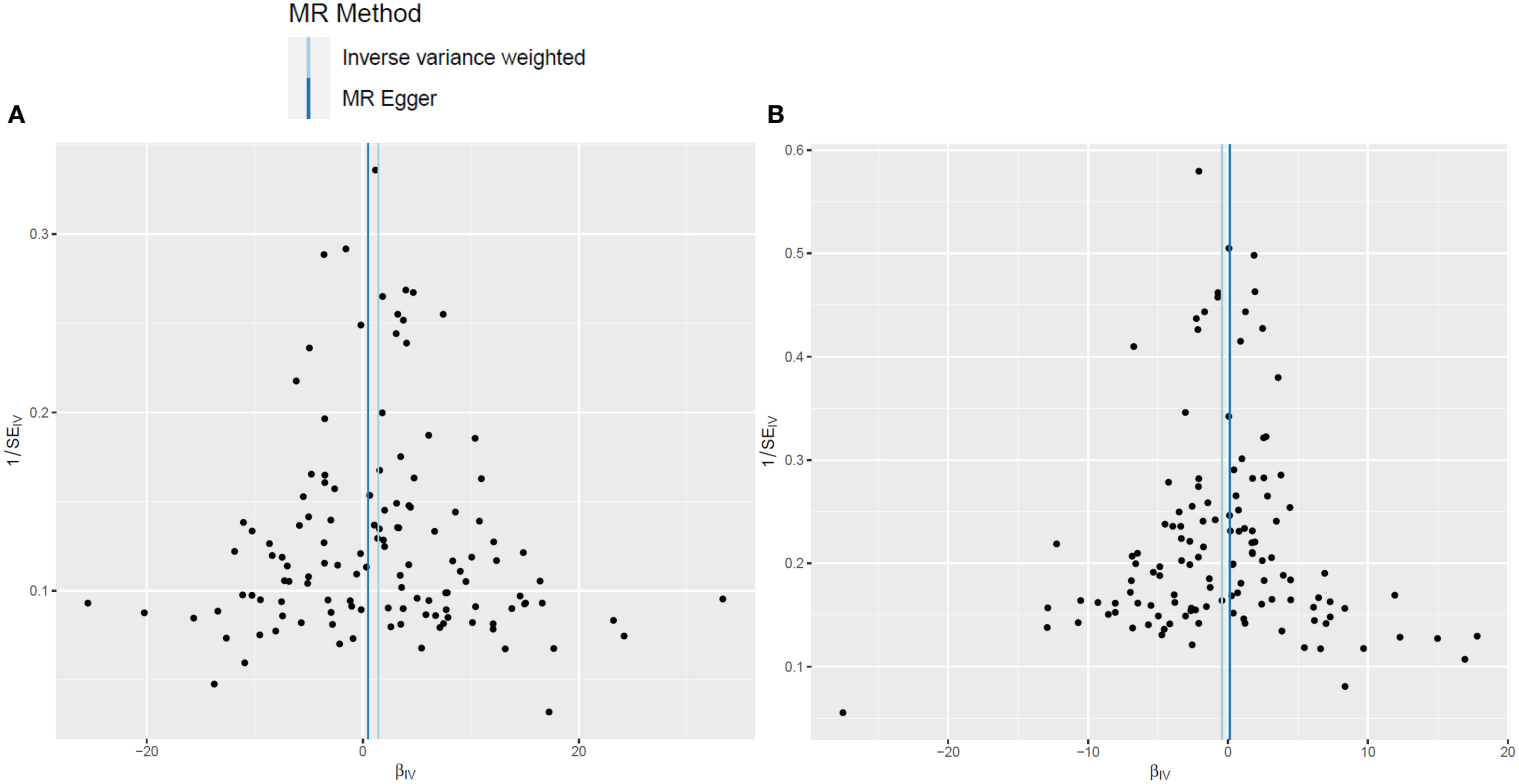
Funnel plots of leukocyte telomere length genetic variants and non-scarring alopecia. **(A, B)**. Funnel plots for androgenetic alopecia **(A)** and alopecia areata **(B)**.

In the two-sample MR analysis, after removal of the SNPs associated with confounding factors and outcomes, along with removal of the SNPs of the incompatible alleles or those that are palindromic with intermediate allele frequencies, a total of 126 LTL-related SNPs that met the three main assumptions of the MR study were retained for further MR analysis. The scatter plots revealed the effects of the above 126 SNPs on LTL and AGA, as well as on AA (**Figures 2A, B**). The funnel plots showed the distribution of the effect of a single SNP (**Figures 3A, B**), while the forest plots visualized the effect of an individual SNP on the estimation of the outcomes (**Supplementary Figures S1A, B**).

**Figure 4:**
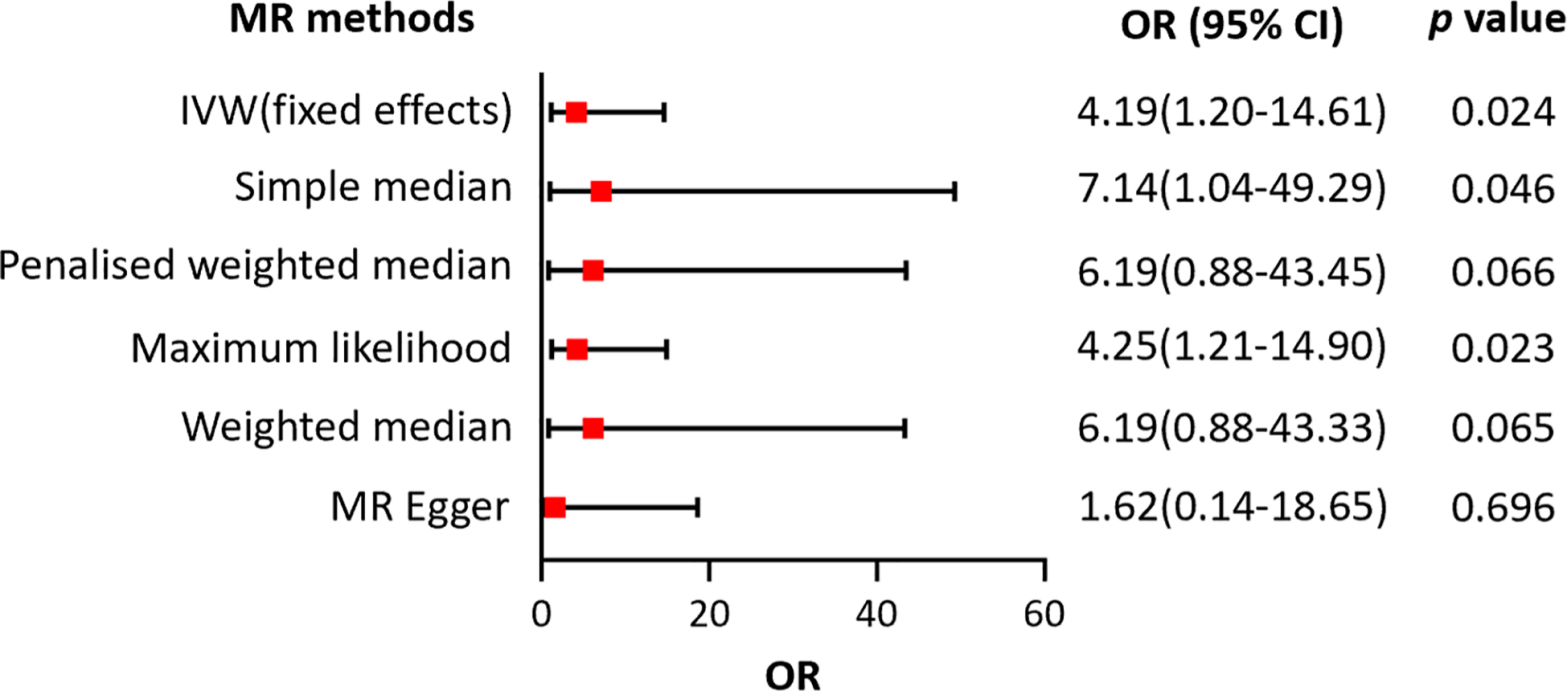
Forest plot of the association between genetically predicted telomere length and androgenetic alopecia. *OR* refers to the change of alopecia risk associated with a one standard deviation (SD) decrease in telomere length. *IVW*, inverse variance weighted; *OR*, odds ratio; *CI*, confidence interval.

The potential causal relationship between LTL and AGA was evaluated using the two-sample MR method. **Table 1** shows that there was no clear evidence of a heterogeneity between the LTL-related SNPs, as the MR-Egger test had a Cochran’s *Q* statistic of 112.0, with a *p*-value greater than 0.05 (MR-Egger method, *p* = 0.771) for AGA. Therefore, the IVW method with a fixed effects model was adopted primarily for causal estimation. The MR analyses suggested a causal relationship between LTL and AGA, and the risk of AGA increased by 3.19 times as the genetically predicted LTL was shortened by one standard deviation in log transformed form under the fixed effects IVW method (OR = 4.19, 95% CI = 1.20–14.61, *p* = 0.024). This causal association was confirmed by maximum likelihood estimation (OR = 4.25, 95% CI = 1.21–14.90, *p* = 0.023) and the simple median method (OR = 7.14, 95% CI = 1.04–49.29, *p* = 0.046) (**Figure 4**). Moreover, the results from the MR-Egger, the weighted median, and the penalized weighted median estimations demonstrated a similar trend of the effect of LTL on AGA, although there was no statistical significance. As mentioned above, the IVW method was adopted as the primary and most efficient method for analysis in this study; therefore, the conclusion that the genetically predicted shorter LTL was associated with an increased risk of AGA was drawn.

**Table 1:** Heterogeneity analysis of the leukocyte telomere length-related genetic variants in the androgenetic alopecia and alopecia areata GWAS datasets.

Exposure	Outcomes	MR methods	*Q* statistic	*Q*-dif	*p*-value
Leukocyte telomere length	AGA	IVW	112.79	125	0.775
		MR-Egger	112.00	124	0.771
	AA	IVW	114.02	125	0.749
		MR-Egger	113.15	124	0.747

*GWAS*, genome-wide association study; *AGA*, androgenetic alopecia; *AA*, alopecia areata; *MR*, Mendelian randomization; *IVW*, inverse-variance weighted; *MR–Egger*, Mendelian randomization–Egger.

**Figure 5:**
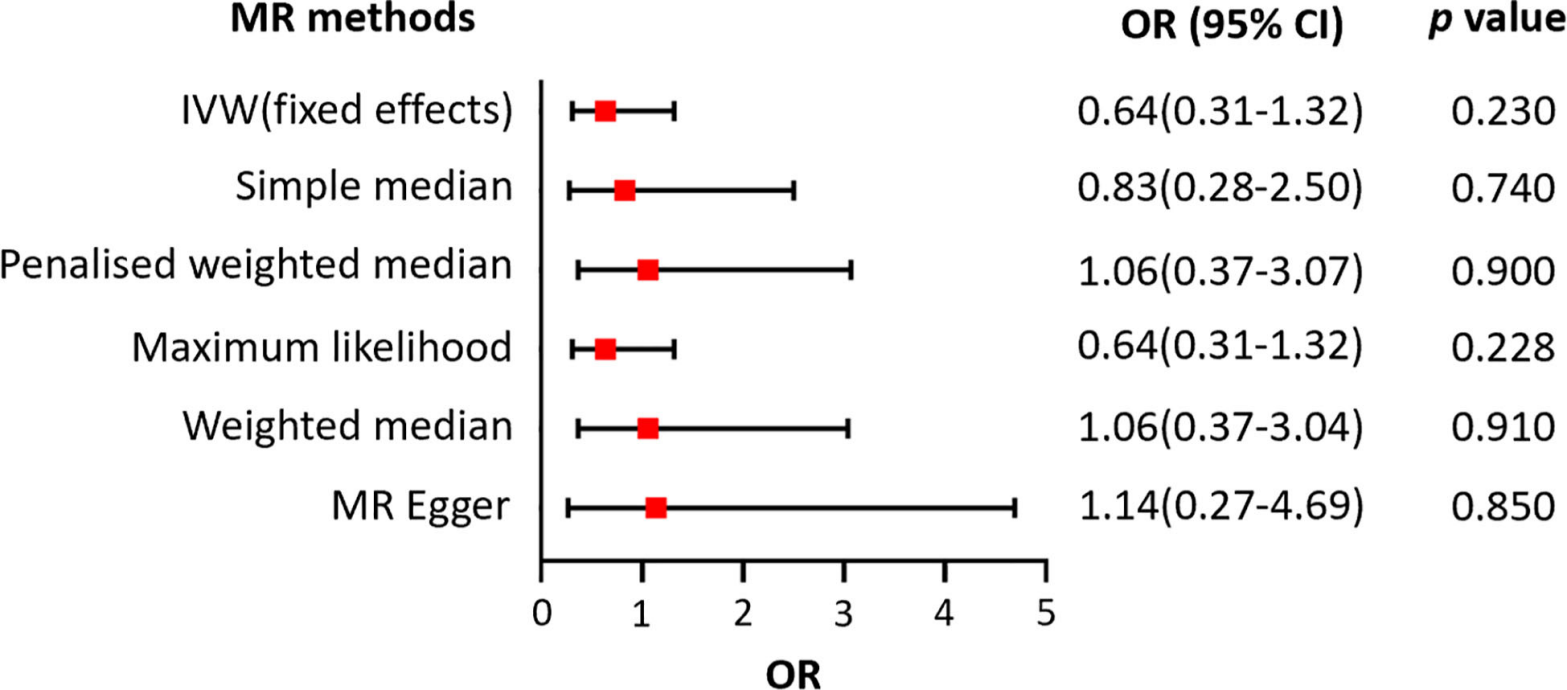
Forest plot of the association between genetically predicted telomere length and alopecia areata.

We also assessed the relationship between LTL and AA using the same MR methods. There was no significant heterogeneity detected, with a *Q* statistic of 113.15 (*p* = 0.747) using the MR-Egger method (**Table 1**). However, the results of the fixed effects IVW, the MR-Egger, the weighted median, the penalized weighted median, and the simple median methods, as well as the maximum likelihood estimation, suggested that there was no clear causal relationship between genetically predicted LTL and risk of AA, with all *p*-values greater than 0.05 (**Figure 5**).

### Sensitivity analysis

No clear evidence for significant statistical heterogeneity was found among the LTL-related SNPs on the effects of AGA and AA, as mentioned previously. In addition, no evidence for directional pleiotropy was found according to the MR-Egger intercept analysis (intercept = 0.028, SE = 0.032, *p* = 0.37), indicating that the exposure–outcome relationship was unlikely to be affected by potential confounding factors through other pathways. The MR-PRESSO analysis suggested no directional pleiotropy and no outliers for the IVs (*p*-global test = 0.795). The leave-one-out analysis yielded comparable results to that of the primary MR studies and indicated that no single SNP has had significant effects on the results, suggesting the robustness and reliability of the MR studies (**Supplementary Figures S2A, B**).

## Discussion

Hair loss has a significant negative impact on body image, social perceptions, and the quality of life of patients and may result in psychological disorders (7, 28, 29). New research works have revealed that complicated factors may contribute to the etiopathogenesis of hair loss, including genetic susceptibility, advanced age, autoimmunological changes, oxidative stress injury, skin microbiome alterations, and epigenetic factors, among other factors (30). To our knowledge AA has affected approximately 2% of the general population, including all sex- and age-based groups (29), while the incidence of AGA has revealed an increasing trend with advanced age. Therefore, this study was performed to assess whether there is an association between non-scarring alopecia, special types of hair loss, and LTL, a well-recognized marker for biological age, rather than chronological age, which may be involved in the pathogenesis of some age-related disorders. In this study, we applied the two-sample MR method to evaluate a potential causal association, which may minimize the bias resulting from confounding factors and reverse causality. The results suggested that, for the European population, the genetically predicted shortened LTL was correlated with a higher risk of AGA, but not of AA.

AA is an autoimmune disorder characterized by non-scarring hair loss on the scalp or any hair-bearing skin. To date, the specific pathogenesis of AA remains unknown, although it has been mainly recognized to be caused by immune disorders and genetic factors. Research studies have revealed that melanocytes may be the trigger point that leads to the hair follicles being attacked by the immune system through oxidative stress injuries or apoptosis (31). On the other hand, different immune cells cooperatively contribute to the progression of AA (32, 33). The infiltration of T helper cells and cytolytic CD8^+^ cells may lead to the disruption of the hair growth cycle. T cells, natural killer (NK) cells, and plasmacytoid dendritic cells surround the lower part of the hair bulb during the anagen and growth phases, and their immunological activities may cause the collapse of the immune privilege of hair follicles and therefore lead to alopecia (7, 34, 35). This process is different from the natural aging process of hair follicles, which may partly account for the result that no association was found between LTL and AA risk.

AGA, a progressive thinning of scalp hair with specific patterns characterized by the hairline receding at the temples (the widow’s peak) and the hair eventually left on the sides and the back of head (the Hippocratic wreath), is now commonly regarded to be correlated with polygenic susceptibility, increased sensitivity to dihydrotestosterone (DHT) of the hair follicle, and chronic scalp inflammation (36). DHT affects hair growth and leads to hair loss through binding to the receptors located in the oil glands of hair follicles, therefore causing the affected follicles to shrink and shortening their anagen cycles, eventually inhibiting the ability of the hair to grow (37, 38). The conversion of testosterone to DHT is catalyzed by 5-α-reductase, an enzyme stored in the oil glands located in hair follicles. Based on the above theories, one recent important treatment strategy emphasizes the suppression of DHT and 5-α-reductase (39, 40); therefore, injuries of the hair follicles may be alleviated and hair loss may be reduced. Previous epidemiological studies suggested that serum DHT and the estradiol (E2) level are correlated with LTL independently of age in men (41), while the testosterone level is not associated with LTL (42).

Observational studies also suggested that, compared with AGA in men, the onset of female androgenetic alopecia (FAGA) occurs in those of more advanced ages and is particularly more common among those undergoing menopause, and the role of androgens remains uncertain in FAGA (1). However, the incidence increasing with age has been observed in both sexes. Advanced age is accompanied by an accumulation of health conditions and the shortening of chromosomal telomere length, signifying an individual’s biological aging. In this study, the results revealed a causal relationship between shortened LTL and a higher AGA risk, which is in accordance with previous observational results. However, aging may be just one of the underlying mechanisms of alopecia, and more studies are still needed to investigate the mechanisms of its onset. One significant strength of the MR method is that the bias resulting from the effects of confounders are removed from the final results. In this study, no SNPs correlated with the DHT concentration were found; therefore, the relationship between telomere length and the risk of AGA was less likely to be affected by the influence of DHT. We also excluded the SNPs related to baldness, hyperthyroidism, and hypothyroidism in order to avoid confounding effects.

As mentioned in other published MR research studies (17, 21, 43), causal inference is considered to be significant if the following criteria were satisfied: 1) the *p*-value using the IVW method is statistically significant (*p* < 0.05); 2) the directions of estimates by the IVW and other methods are all the same (in the current study on AGA, all ORs were greater than 1, prompting the conclusion that the genetically predicted shortened LTL was associated with an increased risk of AGA); and 3) in the sensitivity analyses, the MR-Egger intercept test and the MR-PRESSO global test are not significant (*p* > 0.05). Sensitivity analysis is one of the most important components of MR research, which is designed to detect whether the results are influenced by confounders and bias. In this study, the above criteria were all satisfied; therefore, the conclusion that there might be a causal relationship between LTL and AGA was drawn.

The major strengths of the current MR study are as follows. Firstly, the eligible IVs were obtained from the largest GWAS study from the European Network for Genetic and Genomic Epidemiology (ENGAGE), which satisfied the three main important principles for MR investigation, allowing for reasonable causal estimations. Secondly, the results were valid and consistent in the different MR methods and remained stable in the sensitivity analyses, suggesting the validity and consistency of the causal results. However, this study has several limitations. Firstly, all enrolled individuals in the preliminary GWAS studies were of European ancestry; thus, whether or not the current results could be extended to other populations remains unknown, which needs further investigation. Secondly, we only investigated the relationship between telomere length and the risk of AGA and AA, and due to the lack of summary data for other types of alopecia, the stratification statistics for the degree of AGA, and the stratification data based on sex, further studies were limited. Thirdly, there are also other factors, such as the limitations of the already known phenotype characteristics and LTL determined by genetic factors and that may be affected by environmental factors and potential epigenetic modifications, that have not been thoroughly investigated yet and therefore might contradict the current causal assumptions in future practice. However, these potential effects should not be ignored, and the current conclusions were drawn on the basis of the existing statistics.

Our study suggests that genetically predicted LTL may have a causal effect on the onset of AGA, but not of AA, and that shortened LTL may be one of the risk factors for AGA. The results may offer new insights into the mechanisms of AGA. However, future high-quality randomized controlled studies based on large sample sizes are still needed to further verify this association.

## Conclusion

This MR study suggested that the shortening of the LTL has a negative effect on AGA, while LTL has no association with the risk of AA. Further exploration into the role of LTL on AGA will promote an understanding of the pathogenesis of AGA. A new insight put forward is that preventing the loss of the leukocyte telomere may be a novel target to reduce the risk of AGA.

## Data availability statement

The original contributions presented in the study are partly included in the article/**Supplementary Material**. Further inquiries can be directed to the corresponding author.

## Author contributions

YCL and LY put forward the conception of this study and wrote the manuscript. YCL, STY and MJL performed the statistical analyses. ZJZ and MYL helped revise this manuscript. XRW and MQL collected the related data. All authors contributed to the article and approved the submitted version.

## Supplementary material

Supplementary Figure 1 Forest plot of leukocyte telomere length and risk of androgenetic alopecia **(A)** and alopecia areata **(B)**.

Supplementary Figure 2 Leave-one-out analyses for the effect of leukocyte telomere length and non-scarring alopecia. **(A)** Analysis of leukocyte telomere length and androgenetic alopecia; **(B)** Analysis of leukocyte telomere length and alopecia areata.
