# Supplementary figures and images for: Association between genetically predicted leukocyte telomere length and non-scarring alopecia: A two-sample Mendelian randomization study
Source: Front Immunol. 2023 Jan 30;13:1072573. doi: 10.3389/fimmu.2022.1072573 (PMC9926966; doi:10.3389/fimmu.2022.1072573)

All – MR Egger  
All – Inverse variance weighted

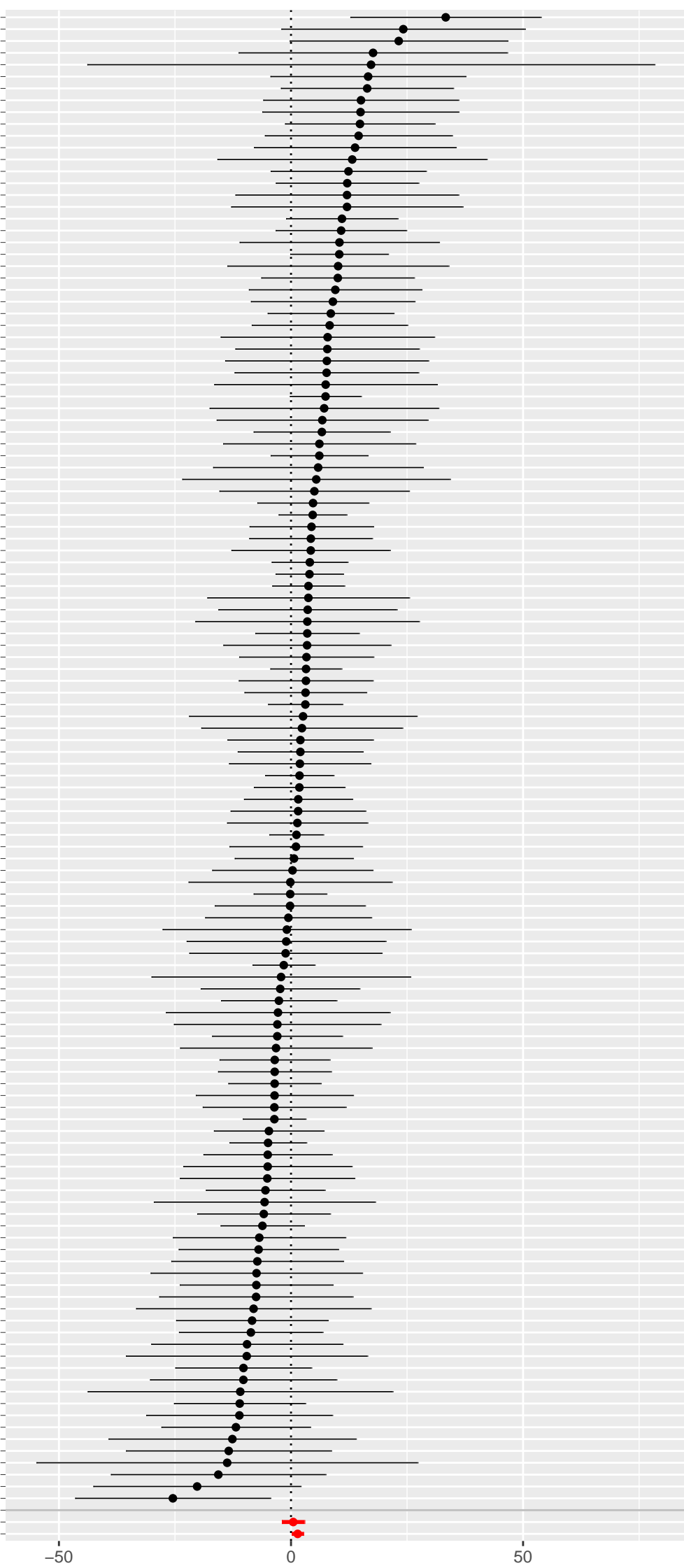

Supplement: Supplementary Figure 1 — Forest plot of leukocyte telomere length and risk of androgenetic alopecia (A) and alopecia areata (B). [file DataSheet_1.pdf]

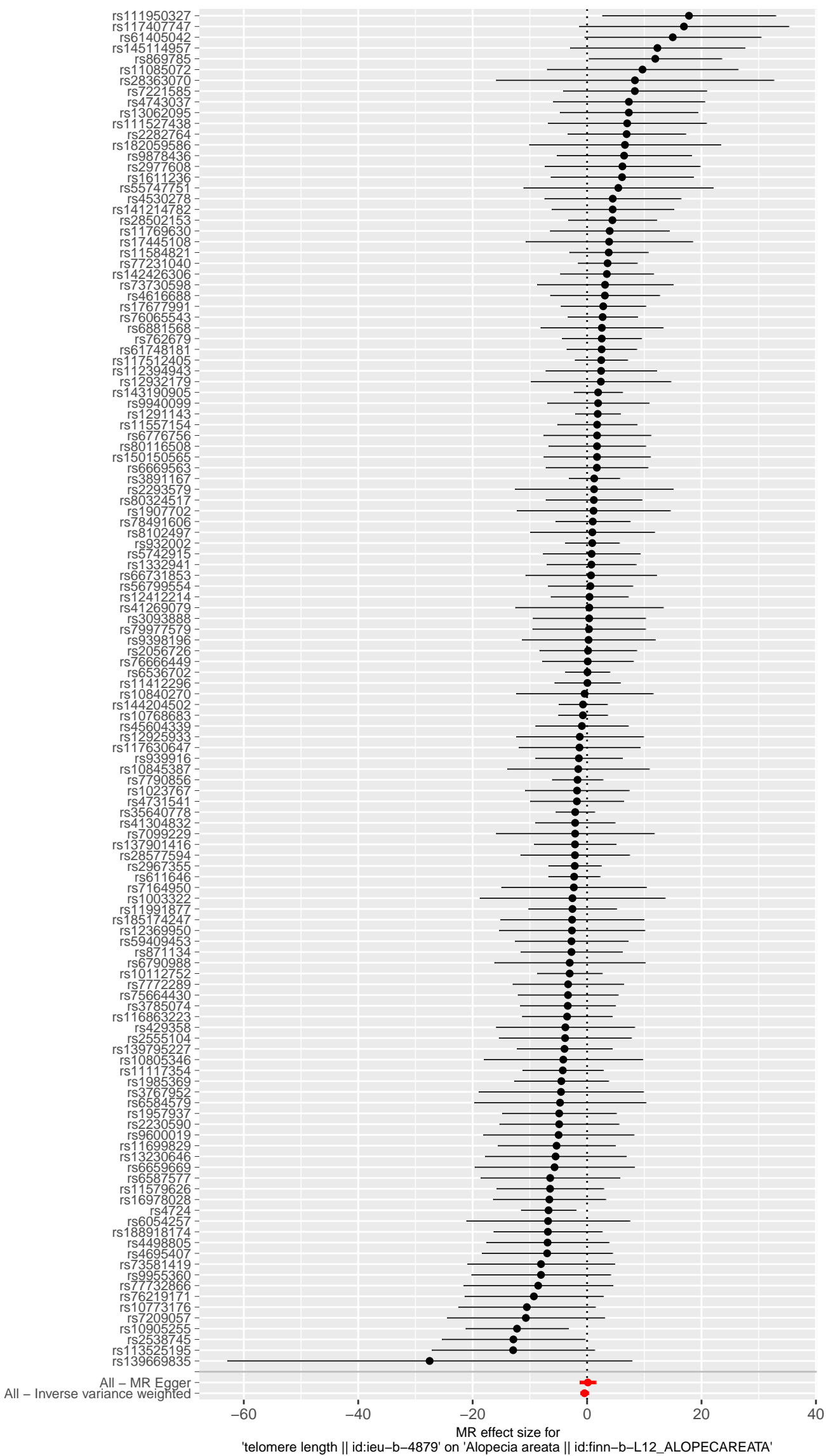

Supplement: Supplementary Figure 2 — Leave-one-out analyses for the effect of leukocyte telomere length and non-scarring alopecia. (A) Analysis of leukocyte telomere length and androgenetic alopecia; (B) Analysis of leukocyte telomere length and alopecia areata. [file DataSheet_2.pdf]
