# Supplementary material for: Association between genetically predicted leukocyte telomere length and non-scarring alopecia: A two-sample Mendelian randomization study
Source: Front Immunol. 2023 Jan 30;13:1072573. doi: 10.3389/fimmu.2022.1072573 (PMC9926966; doi:10.3389/fimmu.2022.1072573)
Supplement: Supplementary file 3 [file DataSheet_3.pdf]

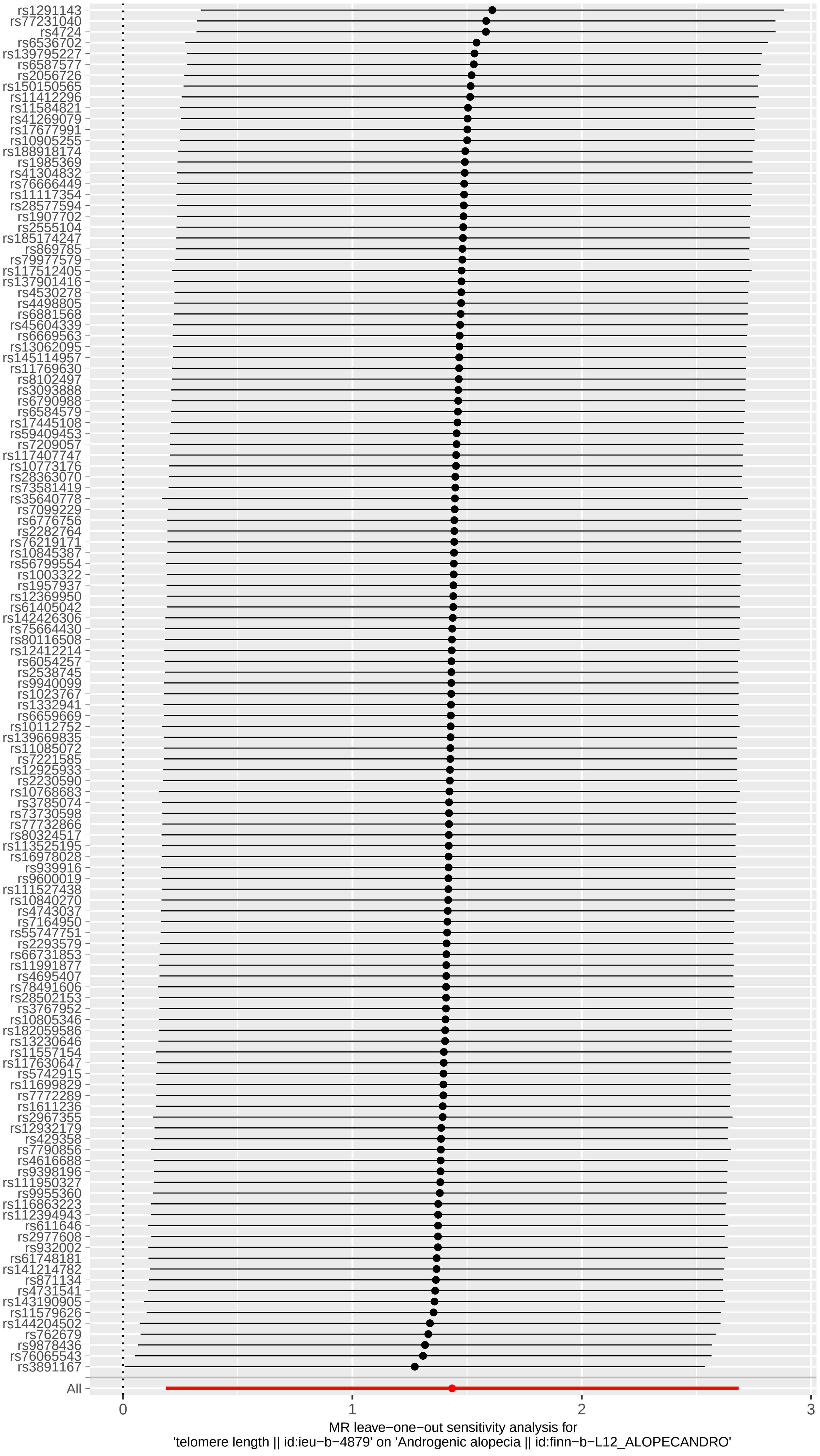
rs1291143
rs77231040
rs4724
rs6536702
rs139795227
rs6587577
rs2056726
rs150150565
rs11412296
rs11584821
rs41269079
rs17677991
rs10905255
rs188918174
rs1985369
rs41304832
rs76666449
rs11117354
rs28577594
rs1907702
rs2555104
rs185174247
rs869785
rs79977579
rs117512405
rs137901416
rs4530278
rs4498805
rs6881568
rs45604339
rs6669563
rs13062095
rs145114957
rs11769630
rs8102497
rs3093888
rs6790988
rs6584579
rs17445108
rs59409453
rs7209057
rs117407747
rs10773176
rs28363070
rs73581419
rs35640778
rs7099229
rs6776756
rs2282764
rs76219171
rs10845387
rs56799554
rs1003322
rs1957937
rs12369950
rs61405042
rs142426306
rs75664430
rs80116508
rs12412214
rs6054257
rs2538745
rs9940099
rs1023767
rs1332941
rs6659669
rs10112752
rs139669835
rs11085072
rs7221585
rs12925933
rs2230590
rs10768683
rs3785074
rs73730598
rs77732866
rs80324517
rs113525195
rs16978028
rs939916
rs9600019
rs111527438
rs10840270
rs4743037
rs7164950
rs55747751
rs2293579
rs66731853
rs11991877
rs4695407
rs78491606
rs28502153
rs3767952
rs10805346
rs182059586
rs13230646
rs11557154
rs117630647
rs5742915
rs11699829
rs7772289
rs1611236
rs2967355
rs12932179
rs429358
rs7790856
rs4616688
rs9398196
rs111950327
rs9955360
rs116863223
rs112394943
rs611646
rs2977608
rs932002
rs61748181
rs141214782
rs871134
rs4731541
rs143190905
rs11579626
rs144204502
rs762679
rs9878436
rs76065543
rs3891167
All
0
1
2
3
MR leave−one−out sensitivity analysis for
'telomere length || id:ieu−b−4879' on 'Androgenic alopecia || id:finn−b−L12_ALOPECANDRO'
