# Supplementary material for: Association between genetically predicted leukocyte telomere length and non-scarring alopecia: A two-sample Mendelian randomization study
Source: Front Immunol. 2023 Jan 30;13:1072573. doi: 10.3389/fimmu.2022.1072573 (PMC9926966; doi:10.3389/fimmu.2022.1072573)
Supplement: Supplementary file 4 [file DataSheet_4.pdf]

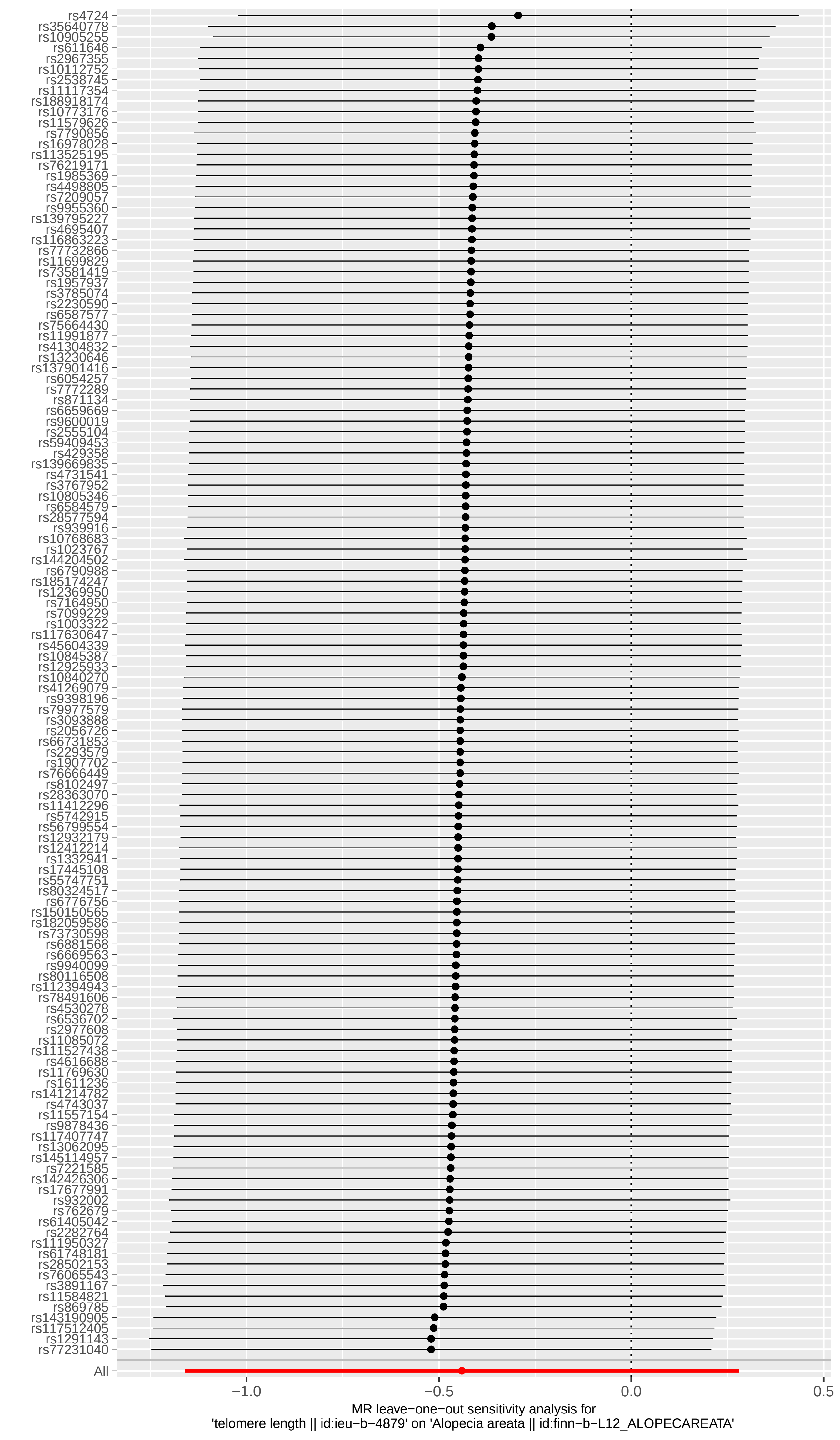
rs4724
rs35640778
rs10905255
rs611646
rs2967355
rs10112752
rs2538745
rs11117354
rs188918174
rs10773176
rs11579626
rs7790856
rs16978028
rs113525195
rs76219171
rs1985369
rs4498805
rs7209057
rs9955360
rs139795227
rs4695407
rs116863223
rs77732866
rs11699829
rs73581419
rs1957937
rs3785074
rs2230590
rs6587577
rs75664430
rs11991877
rs41304832
rs13230646
rs137901416
rs6054257
rs7772289
rs871134
rs6659669
rs9600019
rs2555104
rs59409453
rs429358
rs139669835
rs4731541
rs3767952
rs10805346
rs6584579
rs28577594
rs939916
rs10768683
rs1023767
rs144204502
rs6790988
rs185174247
rs12369950
rs7164950
rs7099229
rs1003322
rs117630647
rs45604339
rs10845387
rs12925933
rs10840270
rs41269079
rs9398196
rs79977579
rs3093888
rs2056726
rs66731853
rs2293579
rs1907702
rs76666449
rs8102497
rs28363070
rs11412296
rs5742915
rs56799554
rs12932179
rs12412214
rs1332941
rs17445108
rs55747751
rs80324517
rs6776756
rs150150565
rs182059586
rs73730598
rs6881568
rs6669563
rs9940099
rs80116508
rs112394943
rs78491606
rs4530278
rs6536702
rs2977608
rs11085072
rs111527438
rs4616688
rs11769630
rs1611236
rs141214782
rs4743037
rs11557154
rs9878436
rs117407747
rs13062095
rs145114957
rs7221585
rs142426306
rs17677991
rs932002
rs762679
rs61405042
rs2282764
rs111950327
rs61748181
rs28502153
rs76065543
rs3891167
rs11584821
rs869785
rs143190905
rs117512405
rs1291143
rs77231040
All
−1.0
−0.5
0.0
0.5
MR leave−one−out sensitivity analysis for
'telomere length || id:ieu−b−4879' on 'Alopecia areata || id:finn−b−L12_ALOPECAREATA'
