# Supplementary material for: Association between genetically predicted leukocyte telomere length and non-scarring alopecia: A two-sample Mendelian randomization study
Source: Front Immunol. 2023 Jan 30;13:1072573. doi: 10.3389/fimmu.2022.1072573 (PMC9926966; doi:10.3389/fimmu.2022.1072573)
Supplement: Supplementary file 5 [file Table_1.docx]

Supplementary Table 1. SNPs used as instrument variables and its association of LTL with AGA and AA.

| **SNP** | **Chr** | **position** | **EA** | **OA** | **SNP-exposure (LTL)** | | | | **SNP-outcome (AGA)** | | | **SNP-outcome (AA)** | | |
| --- | --- | --- | --- | --- | --- | --- | --- | --- | --- | --- | --- | --- | --- | --- |
|  |  |  |  |  | **Beta** | **SE** | | ***p*** | **Beta** | **SE** | ***p*** | **Beta** | **SE** | ***p*** |
| rs1003322 | 22 | 51072289 | A | C | 0.014 | 0.002 | 1.00E-08 | | -0.030 | 0.202 | 0.881 | -0.036 | 0.117 | 0.756 |
| rs10112752 | 8 | 73958718 | A | G | -0.029 | 0.002 | 9.50E-46 | | -0.052 | 0.144 | 0.718 | 0.088 | 0.083 | 0.292 |
| rs1023767 | 8 | 95530969 | A | G | -0.018 | 0.002 | 5.00E-15 | | -0.037 | 0.147 | 0.801 | 0.032 | 0.085 | 0.706 |
| rs10768683 | 11 | 5247791 | G | C | 0.047 | 0.003 | 1.50E-64 | | 0.086 | 0.177 | 0.626 | -0.034 | 0.103 | 0.738 |
| rs10773176 | 12 | 122944713 | G | A | -0.017 | 0.002 | 5.20E-14 | | 0.056 | 0.181 | 0.759 | 0.181 | 0.105 | 0.084 |
| rs10805346 | 4 | 9920347 | C | T | 0.012 | 0.002 | 7.00E-09 | | 0.141 | 0.144 | 0.325 | -0.049 | 0.083 | 0.557 |
| rs10840270 | 11 | 9629553 | G | C | 0.014 | 0.002 | 1.30E-11 | | 0.088 | 0.152 | 0.563 | -0.006 | 0.088 | 0.944 |
| rs10845387 | 12 | 11757743 | A | G | -0.014 | 0.002 | 1.50E-11 | | 0.015 | 0.155 | 0.925 | 0.022 | 0.089 | 0.807 |
| rs10905255 | 10 | 5870267 | T | G | -0.018 | 0.002 | 2.60E-19 | | 0.158 | 0.144 | 0.275 | 0.224 | 0.083 | 0.007 |
| rs11085072 | 19 | 4368142 | T | C | -0.013 | 0.002 | 2.60E-08 | | -0.072 | 0.194 | 0.712 | -0.128 | 0.112 | 0.254 |
| rs11117354 | 16 | 88092092 | C | T | 0.023 | 0.002 | 3.40E-26 | | -0.082 | 0.145 | 0.572 | -0.099 | 0.084 | 0.237 |
| rs111527438 | 17 | 29252703 | C | T | 0.013 | 0.002 | 3.10E-09 | | 0.093 | 0.153 | 0.542 | 0.088 | 0.088 | 0.321 |
| rs111950327 | 16 | 48283993 | C | G | 0.024 | 0.004 | 5.90E-09 | | 0.576 | 0.319 | 0.071 | 0.424 | 0.184 | 0.021 |
| rs112394943 | 3 | 197842892 | C | T | -0.020 | 0.003 | 1.60E-12 | | -0.246 | 0.170 | 0.148 | -0.049 | 0.098 | 0.619 |
| rs113525195 | 14 | 23499321 | A | C | -0.012 | 0.002 | 3.10E-08 | | -0.089 | 0.156 | 0.571 | 0.160 | 0.090 | 0.075 |
| rs11412296 | 15 | 50366116 | T | TA | 0.033 | 0.002 | 1.40E-45 | | -0.117 | 0.169 | 0.489 | 0.002 | 0.097 | 0.984 |
| rs11557154 | 9 | 34107505 | T | C | -0.034 | 0.003 | 1.10E-30 | | -0.163 | 0.211 | 0.438 | -0.060 | 0.122 | 0.620 |
| rs11579626 | 1 | 146741960 | C | A | 0.027 | 0.004 | 1.30E-13 | | 0.394 | 0.218 | 0.071 | -0.171 | 0.126 | 0.175 |
| rs11584821 | 1 | 114419489 | T | C | -0.031 | 0.003 | 3.00E-31 | | 0.146 | 0.185 | 0.431 | -0.116 | 0.107 | 0.280 |
| rs116863223 | 18 | 709396 | A | G | -0.082 | 0.009 | 2.60E-18 | | -0.701 | 0.567 | 0.217 | 0.286 | 0.328 | 0.383 |
| rs11699829 | 20 | 62157200 | A | G | 0.064 | 0.006 | 1.50E-26 | | 0.580 | 0.579 | 0.317 | -0.343 | 0.336 | 0.307 |
| rs117407747 | 7 | 159117178 | T | C | 0.045 | 0.006 | 1.80E-13 | | -0.493 | 0.756 | 0.515 | 0.763 | 0.421 | 0.070 |
| rs117512405 | 20 | 62574274 | A | G | -0.079 | 0.008 | 9.50E-22 | | 0.014 | 0.317 | 0.966 | -0.196 | 0.185 | 0.290 |
| rs117630647 | 7 | 124779510 | A | G | 0.060 | 0.007 | 1.40E-16 | | 0.569 | 0.566 | 0.315 | -0.079 | 0.322 | 0.805 |
| rs11769630 | 7 | 50257703 | A | T | -0.026 | 0.004 | 4.30E-11 | | 0.129 | 0.238 | 0.588 | -0.102 | 0.136 | 0.456 |
| rs11991877 | 8 | 56664524 | A | T | -0.030 | 0.003 | 3.20E-21 | | -0.129 | 0.204 | 0.528 | 0.077 | 0.118 | 0.513 |
| rs12369950 | 12 | 24762109 | C | T | -0.018 | 0.003 | 8.00E-10 | | 0.003 | 0.199 | 0.990 | 0.047 | 0.116 | 0.683 |
| rs12412214 | 10 | 101276256 | A | G | -0.025 | 0.002 | 3.40E-28 | | -0.038 | 0.146 | 0.793 | -0.010 | 0.084 | 0.902 |
| rs1291143 | 20 | 35525640 | C | A | 0.049 | 0.003 | 1.80E-69 | | -0.177 | 0.171 | 0.300 | 0.092 | 0.099 | 0.354 |
| rs12925933 | 22 | 90141355 | C | A | -0.015 | 0.002 | 7.00E-12 | | -0.053 | 0.144 | 0.714 | 0.019 | 0.083 | 0.822 |
| rs12932179 | 8 | 9072085 | G | A | -0.014 | 0.002 | 1.80E-11 | | -0.204 | 0.147 | 0.165 | -0.033 | 0.085 | 0.699 |
| rs13062095 | 8 | 101267385 | C | T | 0.014 | 0.002 | 9.70E-11 | | -0.104 | 0.148 | 0.480 | 0.101 | 0.085 | 0.235 |
| rs13230646 | 11 | 23930316 | C | T | -0.017 | 0.002 | 8.90E-14 | | -0.181 | 0.190 | 0.341 | 0.095 | 0.109 | 0.381 |
| rs1332941 | 12 | 41695100 | G | A | 0.026 | 0.003 | 5.90E-21 | | 0.052 | 0.177 | 0.769 | 0.019 | 0.102 | 0.851 |
| rs137901416 | 4 | 73418095 | A | G | 0.046 | 0.003 | 4.70E-43 | | -0.119 | 0.291 | 0.682 | -0.097 | 0.167 | 0.563 |
| rs139669835 | 11 | 729871 | T | C | -0.061 | 0.011 | 6.10E-09 | | -1.056 | 1.910 | 0.580 | 1.686 | 1.105 | 0.127 |
| rs139795227 | 12 | 92842367 | C | A | 0.060 | 0.009 | 6.70E-12 | | -0.662 | 0.433 | 0.126 | -0.237 | 0.254 | 0.352 |
| rs141214782 | 10 | 78954683 | TTATC | T | -0.025 | 0.003 | 2.00E-13 | | -0.405 | 0.234 | 0.083 | -0.110 | 0.134 | 0.411 |
| rs142426306 | 19 | 62488152 | T | C | -0.050 | 0.005 | 8.70E-21 | | -0.054 | 0.369 | 0.884 | -0.175 | 0.210 | 0.405 |
| rs143190905 | 16 | 62291767 | T | G | -0.072 | 0.004 | 1.60E-85 | | -0.288 | 0.270 | 0.285 | -0.140 | 0.156 | 0.372 |
| rs144204502 | 17 | 76183233 | T | C | -0.101 | 0.009 | 3.40E-28 | | -0.470 | 0.376 | 0.212 | 0.072 | 0.218 | 0.741 |
| rs145114957 | 16 | 94322469 | G | C | 0.027 | 0.005 | 4.60E-08 | | -0.344 | 0.371 | 0.353 | 0.335 | 0.212 | 0.114 |
| rs150150565 | 3 | 708207 | T | C | 0.064 | 0.007 | 6.80E-18 | | -0.757 | 0.522 | 0.147 | 0.111 | 0.303 | 0.715 |
| rs1611236 | 14 | 29748690 | A | G | -0.016 | 0.002 | 6.10E-14 | | -0.221 | 0.178 | 0.214 | -0.098 | 0.102 | 0.336 |
| rs16978028 | 15 | 42070981 | T | A | -0.030 | 0.003 | 8.20E-26 | | -0.127 | 0.261 | 0.626 | 0.198 | 0.150 | 0.188 |
| rs17445108 | 9 | 57082058 | A | G | -0.017 | 0.003 | 2.00E-08 | | 0.136 | 0.218 | 0.532 | -0.065 | 0.126 | 0.605 |
| rs17677991 | 1 | 42032383 | G | C | 0.022 | 0.002 | 4.40E-26 | | -0.123 | 0.146 | 0.400 | 0.063 | 0.084 | 0.454 |
| rs182059586 | 1 | 14652220 | C | T | -0.057 | 0.007 | 4.90E-17 | | -1.010 | 0.844 | 0.231 | -0.378 | 0.487 | 0.438 |
| rs185174247 | 18 | 138914024 | A | G | 0.037 | 0.004 | 1.10E-17 | | -0.500 | 0.421 | 0.235 | -0.098 | 0.238 | 0.682 |
| rs188918174 | 20 | 54473646 | T | C | 0.040 | 0.005 | 1.20E-13 | | -0.338 | 0.337 | 0.315 | -0.276 | 0.195 | 0.157 |
| rs1907702 | 7 | 88955469 | A | G | 0.015 | 0.002 | 5.90E-10 | | -0.235 | 0.177 | 0.185 | 0.017 | 0.103 | 0.870 |
| rs1957937 | 20 | 96181360 | T | A | 0.021 | 0.003 | 1.90E-14 | | 0.007 | 0.185 | 0.970 | -0.102 | 0.106 | 0.338 |
| rs1985369 | 7 | 159119220 | G | A | -0.031 | 0.003 | 3.60E-25 | | 0.183 | 0.228 | 0.423 | 0.140 | 0.131 | 0.285 |
| rs2056726 | 7 | 99780283 | A | G | -0.023 | 0.002 | 7.90E-21 | | 0.234 | 0.171 | 0.171 | -0.004 | 0.099 | 0.968 |
| rs2230590 | 8 | 49936102 | C | T | -0.016 | 0.002 | 3.60E-15 | | -0.055 | 0.145 | 0.707 | 0.077 | 0.084 | 0.359 |
| rs2282764 | 12 | 2255063 | G | A | -0.022 | 0.003 | 9.30E-15 | | 0.013 | 0.205 | 0.950 | -0.155 | 0.118 | 0.188 |
| rs2293579 | 10 | 47440758 | A | G | -0.013 | 0.002 | 3.30E-10 | | -0.131 | 0.157 | 0.404 | -0.016 | 0.091 | 0.864 |
| rs2538745 | 20 | 76310784 | C | T | -0.013 | 0.002 | 3.10E-10 | | -0.031 | 0.143 | 0.831 | 0.167 | 0.083 | 0.043 |
| rs2555104 | 22 | 17841243 | C | A | -0.014 | 0.002 | 6.60E-12 | | 0.155 | 0.143 | 0.277 | 0.054 | 0.082 | 0.515 |
| rs28363070 | 8 | 1415068 | A | G | 0.076 | 0.010 | 3.50E-15 | | -1.039 | 1.584 | 0.512 | 0.632 | 0.935 | 0.499 |
| rs28502153 | 8 | 17469049 | A | C | -0.022 | 0.002 | 1.20E-25 | | -0.095 | 0.147 | 0.517 | -0.096 | 0.085 | 0.260 |
| rs28577594 | 11 | 123895906 | C | G | 0.019 | 0.002 | 5.40E-17 | | -0.140 | 0.158 | 0.375 | -0.040 | 0.091 | 0.663 |
| rs2967355 | 12 | 82200103 | C | A | -0.046 | 0.002 | 4.00E-83 | | -0.143 | 0.189 | 0.450 | 0.099 | 0.108 | 0.359 |
| rs2977608 | 4 | 768253 | C | A | 0.013 | 0.002 | 3.00E-08 | | 0.301 | 0.155 | 0.053 | 0.080 | 0.090 | 0.371 |
| rs3093888 | 11 | 20812951 | A | G | -0.029 | 0.005 | 1.50E-10 | | 0.103 | 0.251 | 0.682 | -0.011 | 0.145 | 0.942 |
| rs35640778 | 12 | 62321128 | A | G | -0.209 | 0.007 | 9.59E-195 | | -0.246 | 0.622 | 0.693 | 0.435 | 0.361 | 0.228 |
| rs3767952 | 10 | 41231032 | A | G | 0.013 | 0.002 | 1.80E-08 | | 0.162 | 0.171 | 0.343 | -0.061 | 0.099 | 0.535 |
| rs3785074 | 19 | 69406986 | G | A | 0.024 | 0.002 | 2.60E-27 | | 0.077 | 0.176 | 0.663 | -0.081 | 0.101 | 0.426 |
| rs3891167 | 16 | 658423 | G | A | -0.043 | 0.002 | 1.20E-70 | | -0.317 | 0.167 | 0.058 | -0.053 | 0.096 | 0.580 |
| rs41269079 | 17 | 45252015 | A | T | 0.015 | 0.003 | 1.70E-09 | | -0.311 | 0.175 | 0.076 | 0.006 | 0.101 | 0.954 |
| rs41304832 | 16 | 62375508 | A | G | 0.061 | 0.009 | 5.00E-11 | | -0.215 | 0.371 | 0.563 | -0.128 | 0.217 | 0.557 |
| rs429358 | 3 | 45411941 | C | T | 0.017 | 0.003 | 3.80E-10 | | 0.262 | 0.186 | 0.161 | -0.066 | 0.107 | 0.538 |
| rs4498805 | 14 | 110910397 | T | G | 0.015 | 0.002 | 5.70E-14 | | -0.109 | 0.143 | 0.444 | -0.104 | 0.082 | 0.206 |
| rs4530278 | 15 | 33752994 | T | G | 0.014 | 0.002 | 1.50E-11 | | -0.132 | 0.146 | 0.368 | 0.062 | 0.084 | 0.461 |
| rs45604339 | 9 | 65543102 | T | C | -0.020 | 0.002 | 4.30E-22 | | 0.060 | 0.146 | 0.680 | 0.019 | 0.084 | 0.826 |
| rs4616688 | 1 | 160042459 | T | G | -0.017 | 0.002 | 4.50E-18 | | -0.175 | 0.146 | 0.231 | -0.054 | 0.084 | 0.522 |
| rs4695407 | 1 | 48843372 | G | A | 0.014 | 0.002 | 1.50E-12 | | 0.111 | 0.143 | 0.439 | -0.099 | 0.082 | 0.231 |
| rs4724 | 18 | 7760397 | A | G | -0.055 | 0.003 | 9.80E-69 | | 0.271 | 0.232 | 0.243 | 0.369 | 0.134 | 0.006 |
| rs4731541 | 20 | 128678236 | G | C | -0.021 | 0.002 | 1.40E-23 | | -0.223 | 0.148 | 0.133 | 0.037 | 0.086 | 0.667 |
| rs4743037 | 7 | 109639970 | T | C | 0.015 | 0.002 | 5.10E-10 | | 0.117 | 0.174 | 0.502 | 0.108 | 0.100 | 0.279 |
| rs55747751 | 20 | 132397351 | A | G | -0.021 | 0.004 | 1.70E-08 | | -0.279 | 0.314 | 0.374 | -0.116 | 0.179 | 0.518 |
| rs56799554 | 7 | 41456413 | G | A | -0.026 | 0.003 | 3.00E-22 | | -0.017 | 0.169 | 0.922 | -0.015 | 0.098 | 0.879 |
| rs5742915 | 7 | 74336633 | C | T | 0.019 | 0.002 | 1.60E-21 | | 0.129 | 0.145 | 0.375 | 0.015 | 0.084 | 0.856 |
| rs59409453 | 8 | 1666218 | G | A | 0.020 | 0.002 | 1.60E-18 | | -0.047 | 0.177 | 0.790 | -0.055 | 0.102 | 0.588 |
| rs6054257 | 12 | 66370 | A | G | -0.014 | 0.002 | 1.10E-08 | | -0.037 | 0.178 | 0.836 | 0.097 | 0.103 | 0.349 |
| rs611646 | 10 | 108177097 | A | T | -0.037 | 0.002 | 3.50E-73 | | -0.138 | 0.146 | 0.344 | 0.083 | 0.084 | 0.323 |
| rs61405042 | 20 | 67200 | T | C | -0.050 | 0.006 | 8.50E-17 | | 0.045 | 0.685 | 0.947 | -0.752 | 0.394 | 0.057 |
| rs61748181 | 22 | 1294166 | T | C | -0.059 | 0.006 | 2.80E-23 | | -0.361 | 0.316 | 0.253 | -0.151 | 0.184 | 0.413 |
| rs6536702 | 8 | 164028105 | A | G | 0.053 | 0.002 | 9.40E-111 | | -0.083 | 0.183 | 0.649 | 0.004 | 0.106 | 0.971 |
| rs6584579 | 8 | 105645725 | G | A | 0.011 | 0.002 | 2.00E-08 | | -0.110 | 0.153 | 0.473 | -0.054 | 0.088 | 0.537 |
| rs6587577 | 11 | 151402045 | G | A | -0.018 | 0.003 | 4.80E-12 | | 0.464 | 0.196 | 0.018 | 0.117 | 0.113 | 0.298 |
| rs6659669 | 12 | 185315067 | T | C | -0.012 | 0.002 | 1.10E-08 | | -0.041 | 0.144 | 0.775 | 0.067 | 0.083 | 0.424 |
| rs6669563 | 4 | 32279629 | A | G | 0.018 | 0.002 | 2.10E-19 | | -0.065 | 0.144 | 0.649 | 0.031 | 0.083 | 0.708 |
| rs66731853 | 11 | 20916238 | A | G | -0.018 | 0.002 | 1.50E-16 | | -0.136 | 0.180 | 0.448 | -0.012 | 0.104 | 0.907 |
| rs6776756 | 12 | 128215821 | A | G | -0.017 | 0.002 | 1.10E-17 | | 0.004 | 0.144 | 0.980 | -0.031 | 0.083 | 0.714 |
| rs6790988 | 10 | 170263320 | G | A | 0.015 | 0.002 | 1.80E-10 | | -0.108 | 0.170 | 0.523 | -0.044 | 0.098 | 0.651 |
| rs6881568 | 19 | 1670265 | A | C | 0.017 | 0.002 | 3.70E-16 | | -0.116 | 0.161 | 0.472 | 0.044 | 0.092 | 0.636 |
| rs7099229 | 16 | 96134685 | A | G | -0.015 | 0.002 | 8.40E-12 | | 0.043 | 0.189 | 0.820 | 0.032 | 0.108 | 0.767 |
| rs7164950 | 17 | 56775385 | G | A | 0.013 | 0.002 | 2.30E-10 | | 0.100 | 0.145 | 0.490 | -0.030 | 0.084 | 0.719 |
| rs7209057 | 16 | 65705530 | A | G | 0.012 | 0.002 | 5.70E-09 | | -0.067 | 0.144 | 0.640 | -0.127 | 0.083 | 0.127 |
| rs7221585 | 3 | 76195153 | T | C | 0.014 | 0.002 | 6.70E-09 | | 0.054 | 0.159 | 0.736 | 0.120 | 0.092 | 0.192 |
| rs73581419 | 14 | 21941148 | T | C | 0.023 | 0.003 | 1.30E-12 | | -0.068 | 0.261 | 0.796 | -0.185 | 0.151 | 0.220 |
| rs73730598 | 15 | 77973 | A | G | 0.027 | 0.004 | 4.70E-10 | | 0.138 | 0.286 | 0.630 | 0.086 | 0.166 | 0.604 |
| rs75664430 | 9 | 8064779 | G | C | -0.024 | 0.002 | 3.60E-24 | | -0.032 | 0.182 | 0.859 | 0.078 | 0.105 | 0.455 |
| rs76065543 | 1 | 74678063 | T | C | 0.034 | 0.003 | 4.20E-32 | | 0.357 | 0.185 | 0.054 | 0.093 | 0.106 | 0.381 |
| rs76219171 | 1 | 50188929 | A | G | 0.036 | 0.004 | 7.80E-17 | | -0.042 | 0.381 | 0.913 | -0.335 | 0.222 | 0.132 |
| rs762679 | 18 | 48885436 | A | T | 0.031 | 0.003 | 1.40E-27 | | 0.341 | 0.190 | 0.074 | 0.080 | 0.110 | 0.469 |
| rs76666449 | 20 | 120904895 | C | T | 0.030 | 0.003 | 8.20E-19 | | -0.148 | 0.209 | 0.478 | 0.003 | 0.120 | 0.978 |
| rs77231040 | 7 | 106280527 | C | G | 0.099 | 0.013 | 2.00E-13 | | -0.609 | 0.455 | 0.180 | 0.355 | 0.261 | 0.174 |
| rs7772289 | 20 | 28674322 | T | G | 0.018 | 0.002 | 1.70E-18 | | 0.146 | 0.150 | 0.331 | -0.058 | 0.087 | 0.502 |
| rs77732866 | 7 | 58979879 | A | G | 0.018 | 0.003 | 9.20E-10 | | 0.104 | 0.205 | 0.613 | -0.152 | 0.118 | 0.198 |
| rs7790856 | 7 | 124459852 | T | C | -0.044 | 0.002 | 1.80E-87 | | -0.142 | 0.171 | 0.409 | 0.073 | 0.099 | 0.459 |
| rs78491606 | 8 | 72891547 | C | A | -0.076 | 0.007 | 1.90E-24 | | -0.265 | 0.432 | 0.539 | -0.075 | 0.251 | 0.765 |
| rs79977579 | 12 | 54694560 | A | C | 0.028 | 0.003 | 2.30E-16 | | -0.197 | 0.247 | 0.426 | 0.009 | 0.142 | 0.948 |
| rs80116508 | 10 | 3650970 | A | G | -0.035 | 0.004 | 2.00E-17 | | -0.054 | 0.262 | 0.836 | -0.061 | 0.152 | 0.688 |
| rs80324517 | 20 | 204031 | A | G | 0.040 | 0.005 | 1.80E-17 | | 0.132 | 0.293 | 0.652 | 0.047 | 0.170 | 0.783 |
| rs8102497 | 22 | 57370055 | A | G | -0.015 | 0.002 | 1.40E-13 | | 0.076 | 0.144 | 0.595 | -0.014 | 0.083 | 0.868 |
| rs869785 | 8 | 24347800 | C | T | -0.015 | 0.002 | 4.40E-12 | | 0.151 | 0.151 | 0.317 | -0.176 | 0.087 | 0.044 |
| rs871134 | 8 | 7044380 | T | C | -0.018 | 0.002 | 1.70E-19 | | -0.222 | 0.144 | 0.123 | 0.050 | 0.083 | 0.547 |
| rs932002 | 11 | 226577306 | T | C | -0.040 | 0.003 | 7.30E-47 | | -0.163 | 0.168 | 0.333 | -0.036 | 0.097 | 0.709 |
| rs9398196 | 12 | 109601554 | G | A | -0.014 | 0.002 | 9.50E-13 | | -0.209 | 0.148 | 0.157 | -0.004 | 0.085 | 0.965 |
| rs939916 | 4 | 202253 | A | G | 0.024 | 0.002 | 6.60E-29 | | 0.076 | 0.162 | 0.641 | -0.035 | 0.094 | 0.710 |
| rs9600019 | 11 | 73317585 | T | C | 0.013 | 0.002 | 2.40E-09 | | 0.086 | 0.148 | 0.561 | -0.063 | 0.085 | 0.458 |
| rs9878436 | 12 | 138244400 | T | C | -0.014 | 0.002 | 1.20E-12 | | -0.478 | 0.150 | 0.001 | -0.093 | 0.086 | 0.281 |
| rs9940099 | 10 | 3613207 | T | G | -0.034 | 0.004 | 3.20E-16 | | -0.064 | 0.262 | 0.806 | 0.400 | 0.414 | 0.335 |
| rs9955360 | 19 | 78008334 | A | C | -0.019 | 0.003 | 2.20E-10 | | -0.316 | 0.204 | 0.122 | 0.153 | 0.118 | 0.194 |

**Abbreviations:** SNP: single-nucleotide polymorphism; LTL: leukocyte telomere length; AGA: androgenetic alopecia; AA: alopecia areata; EA effect allele; OA other allele; SE standard error.
